# Broadly Reactive Human CD8 T Cells that Recognize an Epitope Conserved between VZV, HSV and EBV

**DOI:** 10.1371/journal.ppat.1004008

**Published:** 2014-03-27

**Authors:** Christopher Chiu, Megan McCausland, John Sidney, Fuh-Mei Duh, Nadine Rouphael, Aneesh Mehta, Mark Mulligan, Mary Carrington, Andreas Wieland, Nicole L. Sullivan, Adriana Weinberg, Myron J. Levin, Bali Pulendran, Bjoern Peters, Alessandro Sette, Rafi Ahmed

**Affiliations:** 1 Emory Vaccine Center, Emory University School of Medicine, Atlanta, Georgia, United States of America; 2 Department of Microbiology and Immunology, Emory University School of Medicine, Atlanta, Georgia, United States of America; 3 Centre for Respiratory Infection, National Heart and Lung Institute, Imperial College London, London, United Kingdom; 4 Division of Vaccine Discovery, La Jolla Institute for Allergy and Immunology, La Jolla, California, United States of America; 5 Cancer and Inflammation Program, Laboratory for Experimental Immunology, SAIC Frederick, Inc., Frederick National Laboratory for Cancer Research, Frederick, Maryland, United States of America; 6 Ragon Institute of MGH, MIT and Harvard, Cambridge, Massachusetts, United States of America; 7 Hope Clinic of the Emory Vaccine Center, Emory University School of Medicine, Atlanta, Georgia, United States of America; 8 Division of Infectious Diseases School of Medicine, Emory University School of Medicine, Atlanta, Georgia, United States of America; 9 Departments of Pediatrics, Medicine and Pathology, University of Colorado Anschutz Medical Campus, Aurora, Colorado, United States of America; University of Melbourne, Australia

## Abstract

Human herpesviruses are important causes of potentially severe chronic infections for which T cells are believed to be necessary for control. In order to examine the role of virus-specific CD8 T cells against Varicella Zoster Virus (VZV), we generated a comprehensive panel of potential epitopes predicted *in silico* and screened for T cell responses in healthy VZV seropositive donors. We identified a dominant HLA-A*0201-restricted epitope in the VZV ribonucleotide reductase subunit 2 and used a tetramer to analyze the phenotype and function of epitope-specific CD8 T cells. Interestingly, CD8 T cells responding to this VZV epitope also recognized homologous epitopes, not only in the other α-herpesviruses, HSV-1 and HSV-2, but also the γ-herpesvirus, EBV. Responses against these epitopes did not depend on previous infection with the originating virus, thus indicating the cross-reactive nature of this T cell population. Between individuals, the cells demonstrated marked phenotypic heterogeneity. This was associated with differences in functional capacity related to increased inhibitory receptor expression (including PD-1) along with decreased expression of co-stimulatory molecules that potentially reflected their stimulation history. Vaccination with the live attenuated Zostavax vaccine did not efficiently stimulate a proliferative response in this epitope-specific population. Thus, we identified a human CD8 T cell epitope that is conserved in four clinically important herpesviruses but that was poorly boosted by the current adult VZV vaccine. We discuss the concept of a “pan-herpesvirus” vaccine that this discovery raises and the hurdles that may need to be overcome in order to achieve this.

## Author Summary

Human herpesviruses can cause a wide range of serious infections. They are extremely common and individuals remain latently infected lifelong, with reactivations often causing recurrent or severe disease. T-cells are important in controlling herpesvirus infections and preventing their reactivation, so vaccines that induce T-cells are likely to improve control. Here, we examined human T-cells against VZV that might allow focused vaccine development. We identified a dominant target against which the majority of subjects had mounted a CD8 T-cell response. We found that very similar targets also exist in three other important herpesviruses, HSV-1, HSV-2 and EBV. We showed that CD8 T-cells recognizing the VZV target could also recognize the others and we hypothesized that recurrent encounter with these viruses could boost this common response. In some individuals, immunization with a VZV vaccine did cause activation of these cells, but in most it did not. This reflects the variable efficacy of the currently available VZV vaccine. Our findings suggest that T-cell targets may be shared between herpesvirus species and may therefore contribute to a novel “pan-herpesvirus” vaccine. However, current VZV vaccines cannot reliably stimulate these T-cells and new strategies will be necessary to achieve this goal.

## Introduction

The family Herpesviridae encompasses several highly prevalent human pathogens that cause a spectrum of diseases ranging from mildly symptomatic to severe life-threatening illness [1]. All herpesvirus subfamilies (α, β, and γ) share one important characteristic: the ability to evade the immune response while persisting as latent infections in a state of minimal gene transcription. In many individuals, latent herpesviruses cause no further disease. However, reactivations do occur that lead to considerable morbidity and mortality as well as promoting onward transmission. These events are most frequent in individuals with immunosuppression or immunosenescence [2]. However, asymptomatic reactivation can also occur in immunocompetent individuals, leading to recurrent stimulation of host immunity by herpesvirus antigens [3], [4]. T cells are essential both for recovery from primary herpesvirus infections and prevention of symptomatic reactivation [5]. VZV-specific T cells that secrete Th1 cytokines and exhibit cytolytic activity are detectable following chicken pox [6]. While virus exposure also induces antibodies, the absence of antibodies in children with agammaglobulinemia does not lead to more severe disease [7]. Conversely, the waning T cell immunity that occurs with older age is associated with greater frequency and severity of reactivations [8].

The only herpesvirus vaccines currently available are against VZV. This live attenuated vaccine prevents primary infection in children (i.e. chicken pox) and, when given at high dose, reduces the frequency and/or severity of shingles in elderly adults [9]. The vaccine induces both humoral and cell-mediated immunity [10]–[12], but vaccine-induced immunity can fail and effectiveness in the elderly is relatively poor [13]. The factors underlying this are poorly understood. Rational design of herpesvirus vaccines that elicit optimal protective T cell responses therefore remains an important goal.

However, in order to achieve this, further understanding of the role of human virus-specific T cells during herpesvirus infections is required. In this study, we aimed to comprehensively analyze the breadth of the CD8 T cell response to VZV in the context of the common HLA-A*0201 allele. The VZV genome is large, containing 69 unique open reading frames. This hampers the systematic identification of T cell epitopes and the generation of tools to study them. From VZV, only 7 class I-restricted epitopes from 3 proteins (gI, gB and IE62) have been reported thus far [14]. To address this, we used *in silico* prediction across the entire VZV proteome for epitope mapping. Screening of these candidate peptides in VZV seropositive individuals identified an immunodominant HLA-A*0201-restricted epitope that was conserved with three other herpesviruses. In this study, we characterized the phenotype and function of CD8 T cells that recognized this conserved epitope and also examined the responsiveness of these CD8 T cells to VZV vaccination in humans.

## Results

### Identification of an immunodominant HLA A*0201-restricted VZV T cell epitope

**Figure 1:**
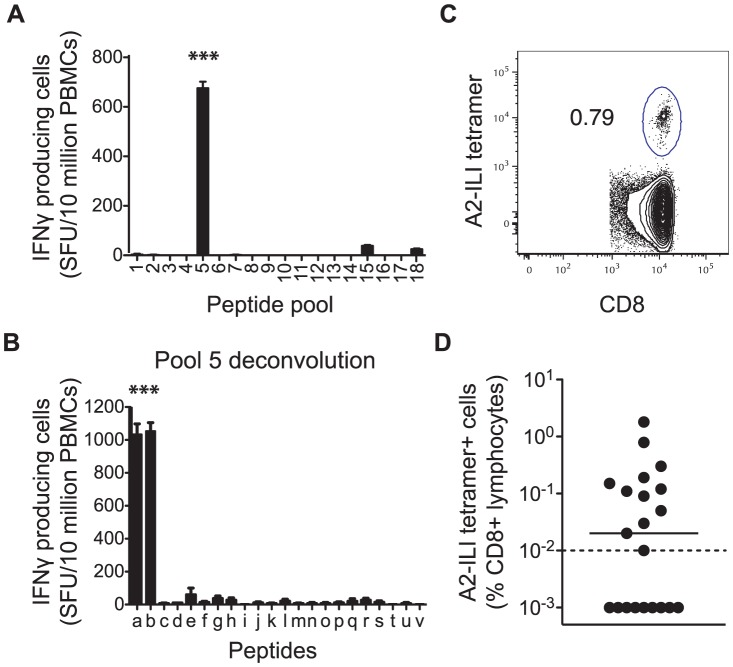
Human CD8 T cells recognize a dominant epitope in the VZV ribonucleotide reductase subunit 2. HLA-A*0201 binding predictions were used to identify candidate VZV-derived epitopes. PBMCs from 21 healthy adult HLA-A*0201+ volunteers were used in IFN-γ ELISpot assays to screen (a) peptide pools and subsequently (b) individual peptides for their ability to stimulate IFN-γ. Asterisks represent p-values<0.001 using Student's t-test. The A2-ILI tetramer was constructed and used to label epitope-specific CD8 T cells. (c) One representative donor is shown. The plot is gated on CD3+CD8+ lymphocytes. The number represents frequency of A2-ILI+ events as a percentage of CD8+ lymphocytes. (d) The frequency of A2-ILI+ CD8 T cells is shown along with the median. The dotted line represents the limit of detectability.

We recruited 21 HLA-A*0201 positive volunteers with a history of primary VZV infection, detectable VZV IgG but no previous VZV vaccination or clinical evidence of recent reactivation (Table 1). The median age was 63 years (range 25–77 years). Epitope predictions in the context of HLA-A*0201 were made using the Immune Epitope Database consensus prediction tool with the complete published VZV sequences (Table S1). The top 0.5% of 9- and 10-mers (367 peptides) were synthesized and peptide pools screened by IFN-γ ELISpot using PBMCs from each subject. In 15/21 subjects, the same peptide pool induced positive responses (Figure 1a). Deconvolution of this pool showed that two candidate peptides (ILIEGIFFV and MILIEGIFFV) from ribonucleotide reductase subunit 2 (RNR2) of VZV strongly induced IFN-γ production (Figure 1b). The predicted MHC-binding of the 9-mer (henceforth called ILI) had 11-fold higher affinity than the 10-mer (Table 2), so this was used to generate the A2-ILI tetramer used to label epitope-specific cells (Figure 1c). Tetramer+ cells were detected in 12/21 subjects with frequencies ranging from 0.01% to 1.8% of CD8 T cells (Figure 1d). ILI was thus identified as an immunodominant HLA-A*0201-restricted class I epitope.

**Table 1:** Summary of demographic data of HLA-A*0201+ subjects.

Number	Age (median + range)	Female %	White %	Black %	Hispanic %
21	63 (25–77)	28.6	80.9	14.3	4.8

**Table 2:** RNR2 epitope homologues in human herpesviruses.

Subfamily	Virus/Organism	Presence of RNR2	Epitope	Predicted affinity IC50 (nM)	Measured affinity IC50 (nM)
α	VZV	+	ILIEGIFFV	1.1	0.03
α	HSV-1	+	ILIEGIFFA	5.2	0.15
α	HSV-2	+	ILIEGVFFA	6.1	0.16
γ	EBV	+	LLIEGIFFI	3.3	0.12
γ	HHV-8	+	LIVEGIYFI*	53	Not done
	Human	+	AAVEGIFFS*	2622	Not done

*Less than 60% homology.

β-herpesviruses do not contain RNR2.

### Phenotypic and functional heterogeneity of ILI-specific CD8 T cells between different individuals

**Figure 2:**
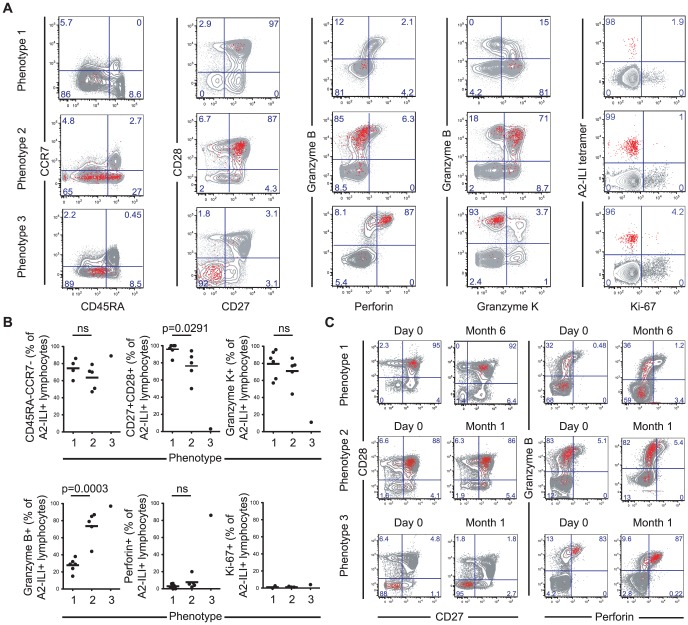
A2-ILI tetramer+ CD8 T cells display distinct phenotypic patterns between individuals. A2-ILI tetramer+ CD8 T cells were co-stained with anti-CD45RA, CCR7, CD27, CD28, granzyme B, granzyme K, perforin, and Ki-67. Phenotypic markers were analysed by flow cytometry. (a) Subjects were categorized according to the combination of phenotypic markers expressed. One representative donor of each phenotype is shown. Numbers represent percentage of CD3+CD8+ lymphocytes. (b) The frequency of A2-ILI+ CD8 T cells in every subject expressing each marker is summarized. The medians are shown and p-values derived from Student's t-test. (c) PBMCs were collected at baseline (day 0) and one month or six months later. A2-ILI tetramer+ CD8 T cells were co-stained with anti-CD27, CD28, granzyme B, and perforin. Phenotypic markers were analysed by flow cytometry. Representative plots from one subject of each phenotype are shown.

To determine the phenotype of these ILI-specific CD8 T cells, we co-stained for memory subset markers; co-stimulatory molecules; and effector molecules. The majority of A2-ILI+ cells were CD45RA-/CCR7- indicative of an effector memory T cell phenotype (Figure 2a & 2b). However, between individuals, these cells displayed marked heterogeneity, which allowed further categorization into one of three phenotypic groups. Phenotype 1 (6/12 subjects) was the least effector-like, expressing high levels of the co-stimulatory receptors CD27 and CD28 with no expression of the cytotoxic molecules perforin or granzyme B; most A2-ILI+ cells of phenotype 2 (5/12 subjects) still expressed CD27 and CD28 and were still negative for perforin, but now expressed granzyme B; finally, phenotype 3 (1/12 subject) described the most effector-like cells with no CD27 and CD28 expression but high perforin and granzyme B (Figure 2a & 2b). We also investigated the expression of granzyme K, a serine protease that marks less differentiated CD8 T cells [15]. This also differed between groups and was inversely associated with granzyme B expression (in keeping with previous reports). In a subset of donors, we went on to examine the differentiation markers KLRG-1 and CD127 (Figure S1a). In all subjects tested, at least half the ILI-specific cells expressed KLRG-1 (a marker commonly expressed on terminally differentiated short-lived effectors), with a trend towards progressively higher expression in phenotypes 2 and 3. A variable proportion expressed CD127 (predominantly expressed on long-lived memory cells) but there was a trend towards more CD127+ cells in phenotype 1 and fewer in phenotype 3. These data were therefore consistent with those used to classify phenotypes 1, 2 and 3, suggesting that ILI-specific cells of phenotypes 2 and 3 were more likely to be terminally differentiated.

Primary VZV infection results in a multi-system disease that includes skin and neurotropic phases and antigen-specific cells may therefore need to localise to a variety of tissues. In most individuals, a major proportion of ILI-specific cells expressed the integrin CD62L, which allows homing to lymphoid organs (Figure S1b). In addition, a variable proportion expressed CCR5, indicating potential for homing to inflamed tissues. With neither marker was there a significant difference in frequency of expression between the 3 phenotypes. A2-ILI+ cells displayed no expression of cutaneous lymphocyte antigen (CLA) but a variable proportion did express the integrin α4β7. Again, there was no correlation with advancing phenotypic group.

One possible explanation for the heterogeneity of phenotype might have been recent or on-going activation, since the combination of markers characteristic of phenotype 3 would also be expected to occur in short-lived effector T cells. We therefore also analysed the expression of Ki-67 to determine whether any of these cells had undergone recent proliferation (Figure 2a & 2b). In a few subjects a minority of A2-ILI+ cells did express Ki-67. However, these never made up more than 5% of the population and there was no evidence that ILI-specific cells of phenotype 2 or 3 were more likely to have had recent proliferative activity. This was supported by analysis of the activation markers CD38 and HLA-DR, neither of which was up-regulated on ILI-specific CD8 T cells (Figure S1c).

In addition, the combinations of markers that segregated the phenotypic groupings did not change over time. Subjects were sampled at intervals between 1 and 6 months from baseline and the frequencies of ILI-specific cells expressing these combinations of markers remained stable (Figure 2c). These data therefore suggested that the ILI-specific memory T cell populations had been observed in a quiescent state and that, while they might express one of several stable phenotypes in any single subject, they were heterogeneous between individuals.

### Functional capacity of ILI-specific CD8 T cells is associated with their phenotype and expression of inhibitory receptors

**Figure 3:**
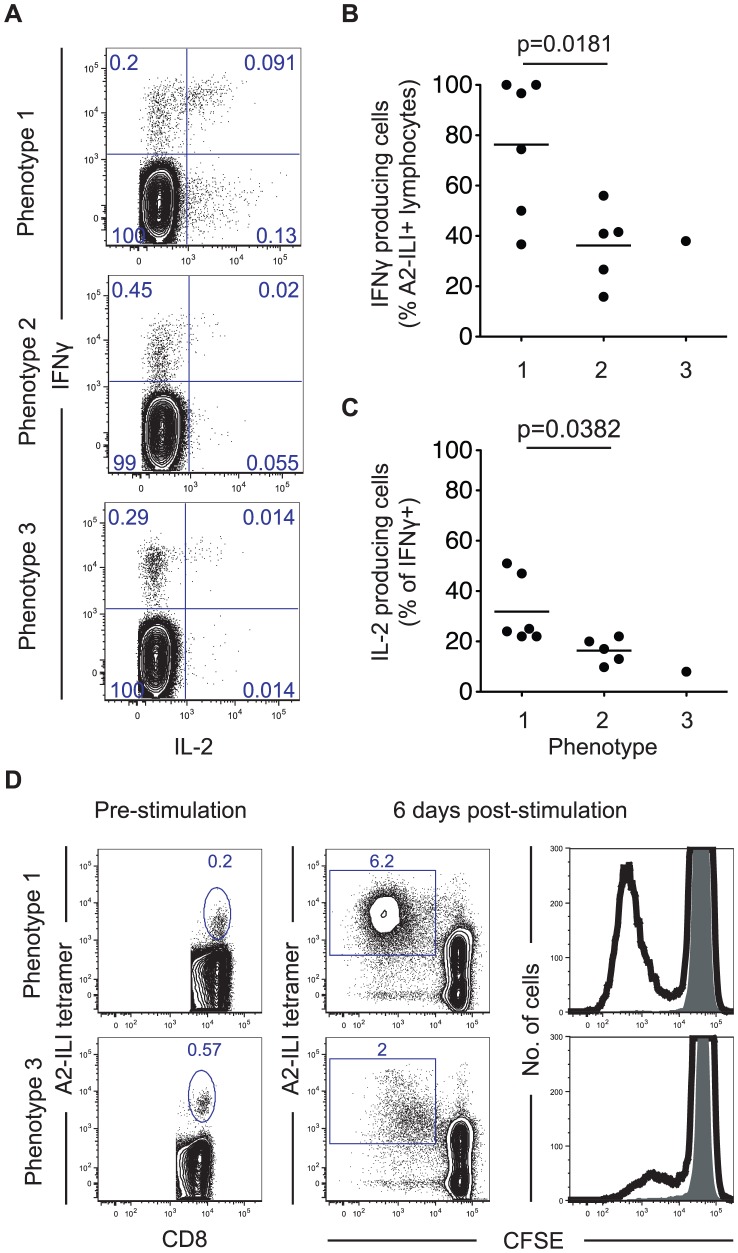
ILI-specific CD8 T cells display functional impairment associated with their phenotype. (a) PBMCs were stimulated with ILIEGIFFV peptide *in vitro* for 6 hours then co-stained for intracellular IFN-γ and IL-2. Numbers represent frequency as a percentage of CD8+ lymphocytes. The frequency of (b) IFN-γ+ cells as a proportion of A2-ILI tetramer+ cells and (c) IL-2 producing cells as a percentage of IFN-γ+ CD8 T cells are shown. P-values are derived from Student's t-test. (d) PBMCs from HLA-A*0201+ volunteers were stained with CFSE and stimulated with ILIEGIFFV peptide *in vitro* for 6 days. A2-ILI tetramer+ cells were then analyzed by flow cytometry. Numbers represent frequency as a percentage of CD8+ lymphocytes. Two representative donors are shown.

In view of their phenotypic differences, we proceeded to examine the functional capacity of ILI-specific CD8 T cells by measuring their ability to produce cytokines and undergo proliferation (Figure 3). Comparing the proportions of A2-ILI+ CD8 T cells capable of producing IFN-γ and IL-2, ILI-specific cells with phenotype 1 had the greatest cytokine producing capacity with a mean of 76% (range 37–100%) expressing IFN-γ (Figure 3b). As the phenotype changed from 1 to 2 and 3, the capacity of ILI-specific cells to produce IFN-γ fell. Furthermore, phenotype 1 ILI-specific cells were also the most polyfunctional, with a mean of 29% (range 22–51%) also staining for IL-2 (Figure 3c). Again, as the phenotype advanced, fewer ILI-specific cells produced this cytokine. In subjects in whom ILI-specific cells were at sufficiently high frequency for the assay, we then analyzed their *in vitro* proliferative capacity. This indicated that ILI-specific CD8 T cells from the individual displaying phenotype 3 were markedly impaired in their proliferation compared with those with phenotype 1 (Figure 3d). Thus the phenotypic and functional patterns displayed by the ILI-specific populations implied that they had been driven, to varying extents between individuals, towards more terminal differentiation with characteristics reminiscent of functional exhaustion.

**Figure 4:**
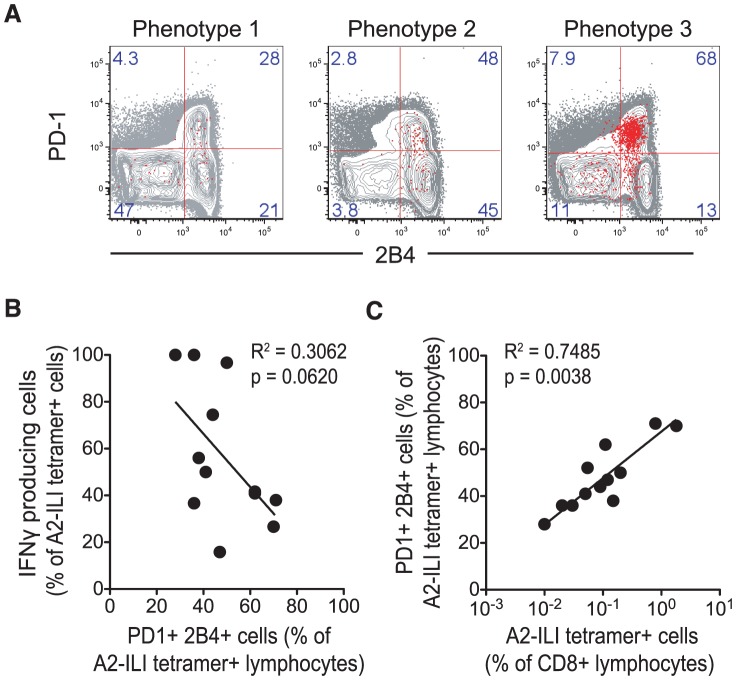
Expression of inhibitory receptors is associated with increased ILI-specific CD8 T cell frequency but impaired functionality. A2-ILI-tetramer+ CD8 T cells were co-stained with anti-PD-1 and 2B4, and analysed using flow cytometry. (a) Representative donors with each phenotype are shown. Numbers represent frequency of events as a percentage of CD3+CD8+ lymphocytes. (b) Non-linear regression and Spearman's rank correlation were used to show the association between the frequency of IFN-γ producing A2-ILI+ cells and their frequency of PD-1 and 2B4 co-expression. (c) Non-linear regression and Spearman's rank correlation were used to show the association between the frequency of A2-ILI tetramer+ CD8 T cells and their expression of both PD-1 and 2B4. P-values for Spearman's rank correlation are shown.

To investigate the potential mechanism underlying this, we examined the association between differentiation phenotype and the expression of the inhibitory receptors PD-1 and 2B4, which are associated with exhaustion and up-regulated on virus-specific CD8 T cells during chronic antigen stimulation [16]. On ILI-specific cells, as the differentiation phenotype progressed, both PD-1 and 2B4 expression increased (Figure 4a). This was associated with a trend towards decreased capacity to produce cytokines such that as the frequency of cells expressing PD-1 and 2B4 increased, the frequency of IFN-γ producing cells fell (Figure 4b).

In chronic viral infections such as HIV, antigen-specific CD8 T cells are abundant, driven by continuous antigenic stimulation via the T cell receptor [17]. However, this increase in the frequency is balanced by increasing expression of inhibitory markers including PD-1, leading to functional exhaustion. Thus, although the frequency of memory T cells is increased, their functionality is restrained. Although herpesviruses do not continually produce antigenic proteins during latent infection, a strong correlation between the size of the population and the frequency of ILI-specific CD8 T cells that co-expressed both inhibitory receptors was seen (Figure 4c). These data imply that recurrent antigen exposure, for example via reactivation, may have driven the proliferation of these cells, thus increasing their frequency but also inducing the expression of inhibitory markers, which in turn affects their functional capacity.

### The epitope recognized by ILI-specific CD8 T cells is conserved between α- and γ-herpesviruses

RNR is one of a number of widely conserved proteins [18], [19]. We therefore hypothesized that the ILI epitope might be well conserved between the human herpesviruses. Indeed, we found that not only was the epitope present in all recorded VZV sequences but that there were also conserved homologues in the α-herpesviruses HSV-1 (ILIEGIFFA) and HSV-2 (ILIEGVFFA), and the γ-herpesvirus EBV (LLIEGIFFI)(Table 2). In contrast, poor homology was seen in the RNR2 of the γ-herpesvirus HHV-8, while the RNR2 gene is absent in the β-herpesviruses CMV, HHV-6 and HHV-7. Furthermore, the homologous epitope from the human RNR has little sequence identity with those of the herpesviruses and therefore unlikely to be responsible for any auto-reactive responses.

**Figure 5:**
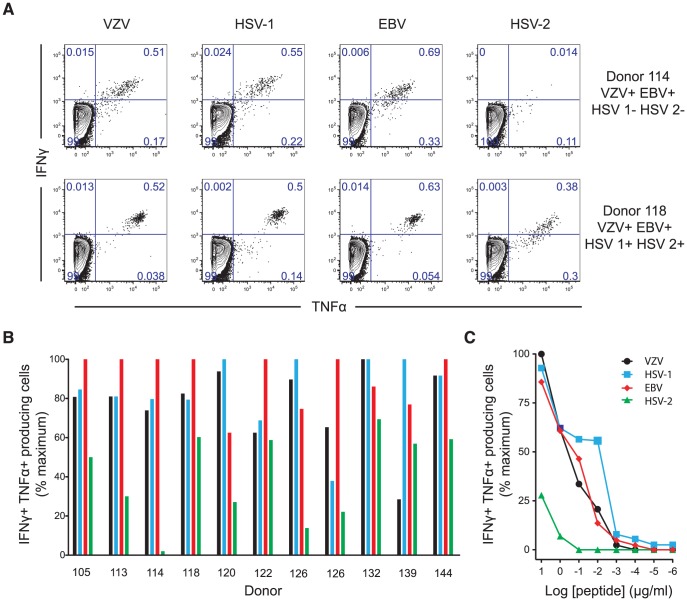
ILI-specific CD8 T cells are broadly reactive, recognizing homologous epitopes conserved between alpha- and gamma-herpesviruses. Homologous epitopes to ILIEGIFFV were identified by sequence similarity in HSV-1, HSV-2 and EBV. (a) The capacity of each epitope to stimulate cytokine production was measured by *in vitro* peptide stimulation. Two representative subjects are shown with their serostatus with respect to the herpesviruses tested. Numbers represent the percentage of CD3+CD8+ lymphocytes. (b) PBMCs from HLA-A*0201+ individuals with detectable ILI-specific responses underwent *in vitro* stimulation with each epitope at a concentration of 10 µg/ml followed by staining with anti-CD3, CD8, IFN-γ and TNF-α. The frequency of cytokine producing cells stimulated by the epitopes from VZV (black), HSV-1 (light blue), EBV (red), and HSV-2 (green) as a percentage of maximal cytokine producing cell frequency is shown. (c) The ability of each epitope to induce cytokine production was assessed by titration of stimulating peptide concentrations. One representative subject (subject 118, seropositive for all herpesviruses tested) is shown. The frequency of cytokine producing cells stimulated by the epitopes from VZV (black), HSV-1 (light blue), EBV (red), and HSV-2 (green) as a percentage of maximal cytokine producing cell frequency is shown.

We tested the recognition of homologous peptides from VZV, HSV-1, HSV-2 and EBV by intracellular cytokine staining (Figure 5a & 5b). In all 11 donors tested, each peptide was capable of stimulating cytokine production. This occurred even when those individuals had no serological evidence of previous infection with the originating virus, with the response to the HSV-1 epitope equivalent to that against the one from VZV even in subjects who were HSV-1 seronegative (Figure 5b & Table 3). Conversely, the reduced response to the HSV-2 epitope occurred even in seropositive individuals. All subjects had been recruited on the basis of seropositivity to VZV and all but three volunteers also had evidence of previous EBV infection (Table 3). In contrast, only 11/21 subjects were positive for HSV-1 IgG and only 3 for HSV-2. Individuals with serological evidence of 3 or more herpesvirus infections were more likely to have detectable A2-ILI+ responses (9/12 subjects) while those with no detectable ILI-specific cells were more likely to have only EBV and/or VZV (6/9 subjects). These data therefore suggest that co-infection with more than two α- or γ-herpesviruses may increase the likelihood of generating a cross-reactive ILI-specific response.

**Table 3:** Donor details and serology.

		A2-ILI tetramer		Serology			
Donor no.	Age	% of CD8+	Phenotype	VZV	HSV-1	HSV-2	EBV
139	25	0.01	1	+	−	−	+
129	29	0.02	1	+	−	−	+
121	26	0.03	1	+	+	−	+
122	77	0.05	1	+	+	−	+
132	70	0.09	1	+	+	−	+
144	39	0.31	1	+	+	−	+
105	73	0.05	2	+	+	−	+
120	73	0.11	2	+	+	−	+
113	33	0.12	2	+	+	−	+
126	66	0.15	2	+	+	−	+
114	74	1.8	2	+	−	−	+
118	64	0.79	3	+	+	+	+
131	26	ND		+	−	−	−
117	28	ND		+	−	−	+
101	30	ND		+	−	−	−
112	37	ND		+	−	−	+
135	62	ND		+	+	−	+
103	63	ND		+	+	+	+
109	69	ND		+	+	−	+
143	70	ND		+	−	+	−
107	71	ND		+	−	−	+

ND – not detected.

Phenotype 1 – CD27+ CD28+ perforin− granzyme B−.

Phenotype 2 – CD27+ CD28+ perforin− granzyme B+.

Phenotype 3 – CD27− CD28− perforin+ granzyme B+.

*In silico* prediction suggested that residues at the anchor motifs of the VZV peptide (position 2 and the C-terminus) conferred optimal binding, while *in vitro* binding measurements showed that all four epitopes displayed extremely high affinities of <0.2 nM (Table 2). However, *in vitro* stimulation of CD8 T cells showed that the HSV-2 epitope was much less effective at stimulating cytokine production than those from VZV, HSV-1 and EBV (Figure 5a, 5b & 5c). Despite the calculated and measured binding affinities, peptide titrations showed that the epitopes from VZV, HSV-1 and EBV induced similar responses in any given individual while the peptide from HSV-2 only stimulated cytokines at its highest concentrations (Figure 5c). We therefore inferred that isoleucine at position 6, absent in the HSV-2 epitope, must be important for efficient TCR recognition.

These data support the hypothesis that herpesviruses are capable of inducing and boosting this epitope-specific response in a cross-reactive manner. Reactivations of one or several of these viruses may cause expansion of this population, increasing its size but also driving the cells towards an increasingly differentiated phenotype.

### Live attenuated VZV vaccine (Zostavax) poorly stimulates proliferation of ILI-specific CD8 T cells

**Figure 6:**
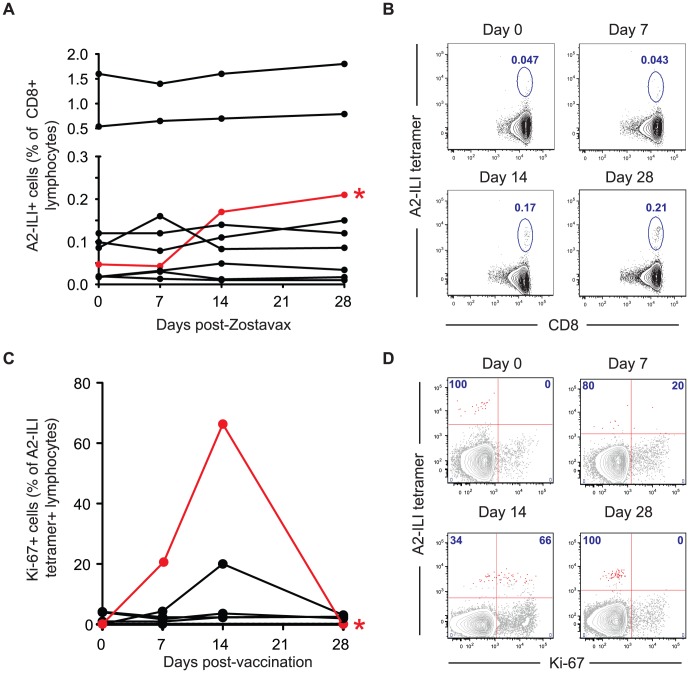
The live attenuated vaccine Zostavax cannot efficiently induce proliferation of ILI-specific CD8 T cells in most individuals. HLA-A*0201+ subjects were immunized with Zostavax. Blood was collected pre-vaccination and at 7, 14 and 28 days post-vaccination. (a) Whole blood was stained with anti-CD3, CD8 and A2-ILI tetramer. The frequency of A2-ILI+ cells post-vaccination in donors with detectable responses is shown. The only responder (subject 105) is marked in red and by an asterisk. (b) FACS plots indicating A2-ILI+ CD8 T cells from the responding donor are shown. Plots are gated on CD3+CD8+ lymphocytes and numbers represent the frequency of A2-ILI+ cells as a percentage of CD8+ lymphocytes. (c) PBMCs were co-stained with anti-Ki-67. The proportion of A2-ILI+ cells expressing Ki-67 post-vaccination in donors with detectable responses is shown. The single responder is marked in red and by an asterisk. (d) FACS plots show the expression of Ki-67 in A2-ILI+ CD8 T cells in the single responder. Plots are gated on CD3+CD8+ lymphocytes and numbers represent the percentage of A2-ILI+ cells with or without Ki-67 expression.

In order to examine the ability of the current VZV vaccine to boost ILI-specific responses, we immunized the study cohort with the live attenuated Zostavax vaccine and tracked the A2-ILI+ response. Following vaccination, the majority of subjects had no detectable change in the frequency of ILI-specific cells irrespective of their pre-vaccination frequency (Figure 6a). A greater than 2-fold increase in epitope-specific cells was seen in only one vaccinee (subject 105, phenotype 2). This individual had one of the lower starting frequencies at 0.05% and incremented to 0.17% at day 14 post-vaccination (Figure 6a & 6b). The increased ILI+ CD8 T cell frequency was associated with up-regulation of Ki-67 in 66% of epitope-specific cells, indicative of proliferation (Figure 6c & 6d). This occurred between 7 and 14 days post-vaccination, with Ki-67 completely down-regulated by day 28. In one additional subject (subject 126), there was evidence of minimal proliferation peaking at day 14 but no overall increase in the frequency of ILI-specific cells (Figure 6c). However, even in the best responder, the overall increase in the frequency of ILI-specific cells was modest despite the higher frequencies being maintained to day 28. Therefore live attenuated VZV vaccine only induced proliferation of ILI-specific cells in a single subject and even in the responding individual, the response was quantitatively poor.

## Discussion

Several T cell epitopes have previously been described that are conserved between HSV-1 and HSV-2 [20], including the epitopes described here [21]. However, it is interesting to find that these conserved epitopes exist more widely in viruses as divergent as the α- and γ-herpesviruses. Here, we have shown that this epitope is, in fact, broadly conserved between 4 different clinically important herpesvirus species despite their sequence divergence. Furthermore, all epitopes were capable of stimulating CD8 T cells even in individuals with no evidence of previous exposure to that virus. Earlier studies have indeed noted that HSV-specific T cells are detectable in a proportion of individuals seronegative for HSV. It was then proposed that these T cells might be induced by subclinical infection or exposure without infection, but our findings suggest that cross-reactivity of T cells may be an alternative explanation [22].

The frequency of ILI-specific CD8 T cells varied widely between individuals. We hypothesize that the number of herpesvirus infections and frequency of reactivations is responsible for these differences. Since herpesviruses are highly prevalent, multiple viruses invariably co-exist within a host and new herpesvirus infections may contribute to further stimulation of cross-reactive T cell populations. Furthermore, although herpesviruses downregulate their transcriptional machinery during latency, chronic expression of some viral genes still occurs [23], and subclinical reactivation has also been widely described. During stress, VZV can be detected in blood or saliva by PCR while HSV-2 often sheds in the absence of genital ulceration [4], [24]. Chronic or periodic antigen exposure may therefore boost T cell numbers over time.

However, in many experimental systems, increasing the number of antigen-specific T cells can also lead to immunopathology and there is increasing evidence in natural infections that this can be controlled by a number of feedback mechanisms. Under conditions of continuous antigen exposure in chronic infections such as HIV, antigen-specific T cells may be driven to proliferate to high frequencies and epitope-specific CD8 T cells identified by tetramer labelling are abundant [17]. However, constitutive expression of inhibitory receptors including PD-1 and 2B4 is also induced by chronic antigen stimulation, leading to reduction in further proliferative capacity and cytokine production [25]–[27]. In tandem with a decrease in co-stimulatory signalling via the down-regulation of CD27 and CD28, immunopathology is restrained despite the higher frequency of potential effector cells. Although continuous production of antigen does not occur in the same way during herpesvirus infections, recurrent antigen stimulation during reactivations may lead to a similar process.

In this context, our data suggest that the differentiation phenotype may act as a biomarker for frequency of reactivation. The phenotypic groups defined by stable expression of differentiation markers in the absence of recent proliferation (as evidenced by Ki-67 expression) may therefore be indicative of antigen exposure history. Furthermore, exhaustion may be one potential mechanism for the impaired T cell function that permits symptomatic reactivations in the elderly. If exhaustion could be reversed, there might be the possibility of enhancing antigen-specific immune responses in this population.

Although T cell immunity is believed to be essential for the control of herpesvirus infections, little is known about the role of T cells in the efficacy of the only currently available herpesvirus vaccines, Zostavax (which protects against shingles) and Varivax (which prevents chicken pox). Both are based on the live attenuated vOka strain of VZV, which has been passaged over 30 times in both human and animal cells. Despite the fact that Zostavax contains 14 times more virus than Varivax (which is administered to children), our data indicate that it does not efficiently stimulate a secondary ILI-specific response in seropositive adults. The explanation for this is likely to be multi-factorial. The numerous mutations that the vOka strain has acquired are likely to have contributed to poor replicative capacity and altered immunogenicity [28]. However, our data also suggest that VZV-specific CD8 T cells in many adults are intrinsically suboptimal, with a balance of co-stimulatory and inhibitory receptors that favors decreased responsiveness to antigen stimulation and impaired functionality. This may partially explain the incomplete protection provided against shingles and why VZV vaccine confers no cross-protection against other herpesviruses despite the presence of this cross-reactive CD8 T cell population in some individuals. Furthermore, VZV, HSV-1 and EBV have all been shown to evade host immunity by interfering with antigen presentation [29]–[31]. Therefore neither existing vaccines nor natural infection are therefore likely to induce cross-reactive T cell responses of sufficient magnitude to provide clinically relevant cross-protection. In addition, it is possible that conserved epitopes such as the ILI homolog are not equally processed and presented in all herpesvirus infections, even when the originating protein (e.g. RNR2) is expressed. The presentation of ILI homologs by HSV-1/2 and EBV in the absence of previous VZV infection was beyond the scope of our study and although CD8+ T cells specific for ILI homologs from HSV-1 and HSV-2 have been described, the VZV serostatus of donors in those studies was not assessed [21]. It therefore remains to be definitively shown whether the cross-reactive CD8+ T cell response demonstrated *in vitro* is effective *in vivo*.

However, in view of the evolutionary relatedness of the herpesvirus subfamilies, we hypothesize that there are still more conserved epitopes to be discovered. Vaccines that induce cross-reactive CD8 T cells might provide protection against clinical disease caused by multiple strains or even species of virus pathogens. The existence of such epitopes could open the way to the development of novel “pan-herpesvirus” vaccines if they can be made to induce responses of sufficient magnitude and functionality. Using the tools we have developed, further identification of the proteins from which similar conserved epitopes are derived may lead us to such a goal.

However, since even natural infection by virulent herpesviruses cannot adequately induce cross-protectivity, development of an effective pan-herpesvirus vaccine will require not only the identification of further cross-reactive epitopes across the major HLA supertypes but also completely new methods to specifically enhance the stimulation of cross-reactive CD8 T cells. In particular, we anticipate that novel adjuncts such as inhibitory receptor blockade will be required to tip the balance of signals that coordinate these responses in the direction of virus-specific T cell activation in order to overcome their relatively functionally impaired state. We anticipate that these strategies as well as advances in the rational design of improved immunogens will ultimately be necessary to achieve a vaccine that effectively protects against the wide array of diseases caused by herpesvirus infection.

## Methods

### Study subjects

All studies were approved by the Emory University Institutional Review Board (IRB #00050285). Study subjects provided written informed consent prior to participation. Clinical information is detailed in Table 1. Twenty-one healthy HLA-A*0201 +ve adults were recruited. Blood was obtained at baseline and multiple time points post-vaccination with the live attenuated Zostavax vaccine (Merck). Study subjects had a history of varicella zoster infection and serologic status against VZV was tested using the VZV IgG ELISAII (Wampole Laboratories, NJ, USA). HLA class I loci were genotyped using the sequence-base typing (SBT) method as recommended by the 13^th^ International Histocompatibility Workshop (Tilanus et al. 2002).

### Bioinformatic analyses and peptide selection

The capacity of all VZV vOka (GI: 26665420, Acc. No. AB097932) derived 9- and 10-mer peptides to bind HLA A*02:01 was predicted using the command-line version of the consensus prediction tool available on the Immune Epitope Database (IEDB) web site (http://tools.immuneepitope.org/main/html/tcell_tools.html). Peptides were selected if they scored in the top 0.5% of predictions for each length. Additional peptides from non-vOka VZV strains (Table S1) were also included if they similarly scored in the top 0.5% of predictions. To assign gene names and locus tags to each peptide, genome sequences were run through an ORF finding algorithm (http://mobyle.pasteur.fr/cgi-bin/portal.py?form=getorf), and the corresponding data copied from the information available for orthologous Dumas proteins. MHC-epitope binding predictions were made with the Stabilized Matrix Method using the tool on the IEDB website. All peptides used in this study were synthesized by Mimotopes (Victoria, Australia) as crude material, and resuspended at 20 mg/ml in 100% DMSO (v/v).

### MHC-peptide binding assays

Quantitative assays to measure the binding of peptides to HLA A*02:01 class I molecules are based on the inhibition of binding of a radiolabeled standard peptide (HBV core 18–27 analogue, FLPSDYFPSV). MHC molecules were purified by affinity chromatography from the EBV transformed homozygous cell line JY, and assays performed, as described previously. Peptides were tested at six different concentrations covering a 100,000-fold dose range in three or more independent assays, and the concentration of peptide yielding 50% inhibition of the binding of the radiolabeled probe peptide (IC50) was calculated. Under the conditions used, where [radiolabeled probe] < [MHC] and IC50 ≥ [MHC], the measured IC50 values are reasonable approximations of the true Kd values.

### PBMC and plasma isolation

PBMCs were isolated using BD Vacutainer CPT tubes, washed, and resuspended in RPMI 1640 with 10% FCS (v/v) for immediate use or frozen in fetal calf serum with 10% dimethyl sulfoxide (v/v) for subsequent analysis. Plasma samples were saved at −80°C for subsequent analysis.

### *Ex vivo* IFN-γ ELISPOT assay

Gamma interferon (IFN-γ) enzyme-linked immunospot (ELISpot) assays were performed using 2×10^5^ PBMC stimulated with peptide pools (10 µg/ml/peptide). Peptide pools yielding positive responses were deconvoluted, by testing individual peptides at 10 µg/ml. After 20 h of incubation at 37°C, plates were developed, and responses were calculated. Positive wells contained ≥20 spot-forming units (SFU)/10^6^ cells and a P value of ≤0.05 using a Student's *t* test in at least 2 experiments.

### Tetramer and antibody staining for flow cytometry

MHC class I tetramer was prepared in-house. Surface staining of T cells was achieved by addition of tetramer to whole blood, incubation for 10 minutes at room temperature, followed by addition of antibody co-stains for 20 minutes. Whole blood was preferred to thawed PBMCs for tetramer labelling due to greater consistency and signal intensity. Following lysis of erythrocytes using BD FACS Lysing solution (BD Biosciences), cells were either fixed using 2% formaldehyde (v/v) or permeabilized using the BD Cytofix/Cytoperm kit for intra-cellular staining. The following antibodies were used for surface and intra-cellular staining: CD3-PerCP, CD8-Horizon V500, CD8-APCH7, Ki-67-FITC, Bcl-2-PE, HLA-DR-Horizon V450, HLA-DR-PerCP, Perforin-FITC, Granzyme B-Horizon V450, CCR7-PE, CD27-Horizon V450, CD27-FITC, CD28-PECy7, CD28-PE (all BD Biosciences) and CD38-PECy7 (eBioscience), Granzyme B-PE (Caltag), Granzyme K-PE (Santa Cruz), CD45RA-FITC (Beckman Coulter), PD-1-PE and 2B4-PerCPCy5.5 (Biolegend). Intra-cellular cytokine staining with IFN-γ-FITC, TNF-α-APC, and IL-2-PerCpCy5.5 (all BD Biosciences) was undertaken after *in vitro* stimulation of PBMCs using peptide for 6 hours. Flow cytometry analysis was performed BD LSRII and BD FACSCanto flow cytometers. Flow cytometry data were analyzed using FlowJo software.

### *In vitro* proliferation assay

PBMC were labeled for 7 minutes with 2.5 µM carboxyfluorescein succinimidyl ester (CFSE, Molecular Probes) in PBS at room temperature. Cold FCS was then added and cells were washed extensively with RPMI 1640 plus 10% FCS. CFSE-labeled cells were incubated with or without the ILIEGIFFV peptide (10 µg/ml) for 6 days. Responding CD8 T cells were subsequently identified by tetramer staining.

## Supporting Information

Figure S1 **Differentiation, homing and activation markers expressed by ILI-specific CD8 T cells.** A2-ILI tetramer+ CD8 T cells were co-stained with (a) anti-CD127 and KLRG-1; (b) CD62L, CCR5, α4β7 and CLA; and (c) CD38 and HLA-DR. Phenotypic markers were analysed by flow cytometry. One representative donor of each phenotypic group is shown. Numbers represent proportion of A2-ILI+ CD8 T cells.
(EPS)

Table S1 **VZV strains used for epitope prediction.** All complete VZV genome sequences published in GenBank were used for *in silico* epitope prediction.
(DOCX)
