# Supplementary material for: Broadly Reactive Human CD8 T Cells that Recognize an Epitope Conserved between VZV, HSV and EBV
Source: PLoS Pathog. 2014 Mar 27;10(3):e1004008. doi: 10.1371/journal.ppat.1004008 (PMC3968128; doi:10.1371/journal.ppat.1004008)
Supplement: Table S1 — VZV strains used for epitope prediction. All complete VZV genome sequences published in GenBank were used for in silico epitope prediction. (DOCX) [file ppat.1004008.s002.docx]

Table S1: VZV strains used for epitope prediction.

| **GI** | **Accession** | **Name** | **Length** | **Strain** |
| --- | --- | --- | --- | --- |
| 9625875 | NC_001348 | Human herpesvirus 3, complete genome. | 124884 | Dumas |
| 291211470 | FV537017 | Modified Microbial Nucleic Acid. | 124884 |  |
| 291211469 | FV537016 | Modified Microbial Nucleic Acid. | 124884 |  |
| 291211468 | FV537015 | Modified Microbial Nucleic Acid. | 124884 |  |
| 291211467 | FV537014 | Modified Microbial Nucleic Acid. | 124884 |  |
| 94482536 | DQ479963 | Human herpesvirus 3 strain 32 passage 72, complete genome. | 125169 | 32 |
| 94482462 | DQ479962 | Human herpesvirus 3 strain 32 passage 22, complete genome. | 125084 | 32 |
| 94482388 | DQ479961 | Human herpesvirus 3 strain 32 passage 5, complete genome. | 124945 | 32 |
| 94482314 | DQ479960 | Human herpesvirus 3 strain 8, complete genome. | 125451 | 8 |
| 94482240 | DQ479959 | Human herpesvirus 3 strain 49, complete genome. | 125041 | 49 |
| 94482166 | DQ479958 | Human herpesvirus 3 strain 36, complete genome. | 125030 | 36 |
| 94482092 | DQ479957 | Human herpesvirus 3 strain 03-500, complete genome. | 125239 | 03-500 |
| 94482018 | DQ479956 | Human herpesvirus 3 strain 22, complete genome. | 124868 | 22 |
| 94481944 | DQ479955 | Human herpesvirus 3 strain 11, complete genome. | 125370 | 11 |
| 94481870 | DQ479954 | Human herpesvirus 3 strain Kel, complete genome. | 125374 | Kel |
| 94481796 | DQ479953 | Human herpesvirus 3 strain SD, complete genome. | 125087 | SD |
| 111184725 | DQ674250 | Human herpesvirus 3 strain NH29_3, complete genome. | 124811 | NH29_3 |
| 90992797 | DQ452050 | Human herpesvirus 3 isolate HHV3_M2DR, complete genome. | 124770 |  |
| 46981482 | AY548170 | Human herpesvirus 3 strain MSP, complete genome. | 124883 | MSP |
| 46981409 | AY548171 | Human herpesvirus 3 strain BC, complete genome. | 125459 | BC |
| 26665422 | AB097933 | Human herpesvirus 3 DNA, complete genome, strain: Oka, sub_strain: pOka. | 125125 | Oka |
| 26665420 | AB097932 | Human herpesvirus 3 DNA, complete genome, strain: Oka, sub_strain: vOka. | 125078 | Oka |
| 157965723 | EU154348 | Human herpesvirus 3 strain SVETA, complete genome. | 124813 | SVETA |
| 66866043 | DQ008355 | Human herpesvirus 3 strain VariVax, complete genome. | 124815 | VariVax |
| 66865971 | DQ008354 | Human herpesvirus 3 strain VarilRix, complete genome. | 124821 | VarilRix |
| 91980297 | DQ457052 | Human herpesvirus 3 strain CA123, complete genome. | 124771 | CA123 |
| 83721806 | AJ871403 | Human herpesvirus 3 (HHV-3), complete genome, isolate HJ0. | 124928 |  |
| 59989 | X04370 | Human herpesvirus 3 (strain Dumas) complete genome. | 124884 | Dumas |
